# Immunomodulatory Role of Tenascin-C in Myocarditis and Inflammatory Cardiomyopathy

**DOI:** 10.3389/fimmu.2021.624703

**Published:** 2021-02-22

**Authors:** Kazuko Tajiri, Saori Yonebayashi, Siqi Li, Masaki Ieda

**Affiliations:** Department of Cardiology, Faculty of Medicine, University of Tsukuba, Tsukuba, Japan

**Keywords:** tenascin-C, myocarditis, inflammatory cardiomyopathy, autoimmunity, extracellular matrix

## Abstract

Accumulating evidence suggests that the breakdown of immune tolerance plays an important role in the development of myocarditis triggered by cardiotropic microbial infections. Genetic deletion of immune checkpoint molecules that are crucial for maintaining self-tolerance causes spontaneous myocarditis in mice, and cancer treatment with immune checkpoint inhibitors can induce myocarditis in humans. These results suggest that the loss of immune tolerance results in myocarditis. The tissue microenvironment influences the local immune dysregulation in autoimmunity. Recently, tenascin-C (TN-C) has been found to play a role as a local regulator of inflammation through various molecular mechanisms. TN-C is a nonstructural extracellular matrix glycoprotein expressed in the heart during early embryonic development, as well as during tissue injury or active tissue remodeling, in a spatiotemporally restricted manner. In a mouse model of autoimmune myocarditis, TN-C was detectable before inflammatory cell infiltration and myocytolysis became histologically evident; it was strongly expressed during active inflammation and disappeared with healing. TN-C activates dendritic cells to generate pathogenic autoreactive T cells and forms an important link between innate and acquired immunity.

## Introduction

Myocarditis is an inflammatory disease of the myocardium. It represents a public health challenge worldwide, as it is one of the leading causes of dilated cardiomyopathy, particularly in young, previously healthy individuals (1). Myocarditis can be triggered by a variety of infectious and non-infectious agents (2, 3), and the subsequent autoimmune response is thought to contribute to the disease progression to inflammatory cardiomyopathy (4, 5).

Tenascin-C (TN-C) is a non-structural extracellular matrix (ECM) expressed during embryonic development in the heart, but is not present in the normal adult heart (6). In tissue injury, inflammation, or active remodeling, TN-C is re-expressed in a spatiotemporally restricted manner (6). Recently, TN-C has gained attention as a local regulator of inflammation through various molecular mechanisms (7). Several animal studies have revealed that TN-C is involved in autoimmune disorders, including myocarditis, arthritis, glaucoma, and encephalomyelitis (8–11). In autoimmune myocarditis, TN-C activates dendritic cells (DCs) to generate pathogenic autoreactive T cells and forms an important link between innate and acquired immunity (9). In this mini review, we discuss the mechanistic insights into the development of myocarditis and its progression to inflammatory cardiomyopathy and focus on the role of TN-C in their pathology.

## Triggers of Myocarditis

Myocarditis can occur in association with a wide spectrum of infectious agents (such as viruses, bacteria, and protozoans), systemic immune-mediated diseases, toxic substances, and drugs (such as immune checkpoint inhibitors) (2, 3, 12, 13). Viruses have been implicated as the leading trigger of myocarditis, with cardiotropic viruses (such as coxsackievirus B3 [CVB3] and adenoviruses), vasculotropic viruses (such as parvovirus B19), and lymphotropic viruses (such as human herpesvirus 6), which are common agents identified in the myocardium of patients with myocarditis/dilated cardiomyopathy (DCM) (14–16). However, the etiologic role of the viruses detected in myocarditis patients is not evident (17). For example, a high prevalence of parvovirus B19 has been observed in hearts both with (18) and without myocarditis (19). Thus, the causative or associative link between individual viral infections and the pathogenesis of myocarditis is still under investigation (17). In Latin America, infection by the protozoan parasite *Trypanosoma cruzi* is the most common cause of inflammatory heart disease (17).

Animal models of virus-induced myocarditis with CVB3 infection have been used to study how viruses trigger myocarditis. The pathogenesis of CVB3-induced myocarditis involves both viral cytotoxicity and subsequent host immune responses (2). Initially, CVB3 enters cardiomyocytes by binding to the coxsackievirus-adenovirus receptor, and causes direct cytotoxicity to the myocardium within three to four days post-infection (16). During the early stage of CVB3 infection, innate immune cells are activated through pattern recognition receptors, such as toll-like receptors (TLRs), and produce pro-inflammatory cytokines, such as interferons (IFNs), interleukin (IL)-1β, IL-6, IL-8, and tumor necrosis factor-α (16, 20). Subsequently, antigen-specific responses in adaptive immune cells are induced, eliminating the virus by up to 14 days post-infection (16, 20). However, even after viral clearance, a subset of individuals may develop chronic myocardial inflammation with virus-triggered uncontrolled immune response and the expansion of cardiac-autoreactive T cells, leading to inflammatory cardiomyopathy.

Infection with the novel pathogen severe acute respiratory syndrome coronavirus 2 (SARS-CoV-2) may trigger myocarditis both directly and indirectly (14, 21). Several studies have suggested that the immune response triggered by the virus is the major cause of cardiomyocyte injury, rather than direct virus-mediated cytotoxicity (15, 22). In a recent series of hospitalized coronavirus disease 2019 (COVID-19) cases caused by SARS-CoV-2 infection, acute cardiac injury with serum troponin elevation occurred in 7% to 27% of patients, and elevated troponin levels were associated with increased mortality in patients with COVID-19 (23–25). However, elevated troponin can be caused not only by myocarditis but also by other heart diseases, such as ischemic heart disease, Takotsubo syndrome, or secondary cardiac injury due to systemic inflammation and hypoxemia due to respiratory dysfunction (14, 26). Recently, autopsies of 21 patients who died from COVID-19 identified multifocal lymphocytic myocarditis in three cases (14%) (26). Viral entry to cardiac cells using angiotensin converting enzyme 2 may directly induce myocarditis (27). However, the exact mechanism of SARS-CoV-2-induced myocarditis is currently unknown, and further investigations are required.

## Autoimmune Myocarditis and Inflammatory Cardiomyopathy

Myosin heavy chain α isoform (MyHC-α) represents a major cardiac autoantigen. MyHC-α immunization with immune adjuvants or the injection of MyHC-α-loaded DCs can induce autoimmune myocarditis in mice (28, 29). MyHC-α reactive T cells have been found in patients with myocarditis and, interestingly, in healthy subjects (30), suggesting that this may be due to impaired T cell tolerance mechanisms. In the thymus, most autoreactive T cells are eliminated through central immune tolerance or negative selection. In this process, the presentation of self-peptides by antigen-presenting medullary thymic epithelial cells is crucial for determining the fate of developing T cells. Importantly, unlike other cardiac antigens, MyHC-α is not expressed in thymic cells in either mice or humans. Therefore, a lack of central T cell tolerance to this protein allows MyHC-α-reactive T cells to escape negative selection and enter the peripheral circulation (30). MyHC-α-reactive T cells were markedly increased in myocarditis, and adoptive transfer of these cells induced myocarditis in the recipients, demonstrating the effector function of MyHC-α-reactive T cells (30). Although frequency is low, MyHC-α-reactive T cells are present in the periphery of healthy individuals (30), suggesting that peripheral immune tolerance is crucial to prevent these self-reactive T cells from inducing autoimmune myocarditis (5, 31, 32). The mechanism of peripheral immune tolerance is complicated, and immune checkpoints, including cytotoxic T-lymphocyte antigen 4 (CTLA-4) and programmed cell death protein 1 (PD-1)/PD ligand 1 (PD-L1), play important roles in maintaining peripheral tolerance to cardiac antigens (33). After viral infection, MyHC-α-specific CD4^+^ T cells expand, likely because of molecular mimicry (epitope cross-reactivity) or epitope spreading (self-antigen exposure from cardiomyocytes upon viral damage), and contribute to post-infectious myocarditis (34).

Until now, the role of gut bacteria in cardiac autoimmunity was unclear. However, recently, Gil-Cruz et al. (35) demonstrated that the commensal gut microbe *Bacteroides thetaiotaomicron* (*B. theta*) triggers a cross-immune response against the bacterial protein β-galactosidase and MyHC-α, causing inflammatory cardiomyopathy. B-galactosidase produced by *B. theta* has sequence homology to MyHC-α and induces proliferation and T helper 17 (Th17) polarization of MyHC-α-specific CD4^+^ T cells. Additionally, antibiotic therapy prevents lethal consequences. Patients with myocarditis have higher anti-*B. theta* antibody and circulating T cells from the patients show significantly higher IFN-γ production capacity against both MyHC-α and β-galactosidase than healthy subjects. These results suggest that targeting the microbiome could become a new therapeutic strategy.

In contrast to the well-defined cardiac antigen-specific T-cell responses, our understanding of the role of heart non-specific CD4^+^ T cells in myocarditis is limited. Recently, Zarak-Crnkovic et al. (36) demonstrated in a proof-of-concept study that heart non-specific effector T cells did not affect the severity of myocarditis, but protected the heart from adverse post-inflammatory fibrotic remodeling and cardiac dysfunction in the chronic stage. Moreover, bystander activation of effector T cells suppressed the myofibroblast phenotype of mouse and human cardiac fibroblasts (36), suggesting a dynamic and complex role of effector T cells and the interplay between T cells and fibroblasts in autoimmune myocarditis.

## TN-C in Myocarditis and Inflammatory Cardiomyopathy

In the heart, TN-C is transiently expressed at several important stages during embryonic development, but TN-C-deficient mice do not show a clear phenotype (37). TN-C is rarely expressed in normal adult hearts but is upregulated under pathological conditions with tissue injury, tissue repair/regeneration, and inflammation (38, 39), including myocarditis (40, 41), DCM (42), rheumatic heart disease (43), myocardial infarction (44, 45), hypertensive heart disease (46), and Kawasaki disease (47). Serum TN-C levels appear to be useful biomarkers for assessing disease activity and predicting disease prognosis. High serum TN-C levels are a significant independent predictor for cardiac events and have an incremental predictive power with brain natriuretic peptide (BNP) in both myocardial infarctions and DCM (48, 49). BNP is secreted from cardiomyocytes in the ventricles in response to stretching caused by increased wall tension and is broadly used as a marker for the diagnosis and treatment of heart failure (50, 51). On the other hand, fibroblasts are a major source of TN-C in the pathological heart (40, 52, 53). The combination of the two biomarkers may more accurately reflect the pathological condition of the entire heart than a single biomarker (54).

The expression of TN-C is detectable in the heart before inflammatory cell infiltration and myocytolysis become histologically apparent, persists during active inflammation, and is no longer present prior to mature collagen deposition in the healing phase in a mouse model of experimental autoimmune myocarditis (5). A major source of TN-C in the pathological heart consists of residential interstitial cells, primarily fibroblasts; however, precardiac mesodermal cells, a special population of cardiomyocytes in embryonic hearts, and several cell lines of cardiomyocytes also have the potential to produce TN-C (54). Its expression level reflects the activity of myocardial inflammation (40). We previously investigated the immunomodulatory effect of TN-C in experimental autoimmune myocarditis. TN-C-deficient mice were protected from severe myocarditis with lower Th17 cell infiltration to the heart compared to wild-type mice (9). Th17 cells are closely associated with autoimmunity, and IL-17-producing Th17 cells play a major role in the initiation and development of myocarditis (2, 55, 56). In human myocarditis/inflammatory cardiomyopathy, the Th17 immunophenotype is characterized by elevated Th17 levels with increases in the Th17-related cytokines IL-6, IL-1β, transforming growth factor-β1, IL-23, and granulocyte-macrophage colony-stimulating factor (GM-CSF) (57). Moreover, patients with severe heart failure have greater proportions of Th17 than those with low severity heart failure (57). IL-6 is a key cytokine that differentiates naïve CD4^+^ T cells into Th17 cells (58). The stimulation of DCs with exogenous TN-C produces high levels of IL-6 (9). Naive CD4^+^ T cells co-cultured with TN-C-stimulated DCs differentiate into Th17 cells, and the IL-6 blockade inhibits Th17 polarization (9). In addition, TN-C-stimulated DCs produce high levels of IL-1β and GM-CSF, which facilitate Th17 generation and maintenance (59, 60). Taken together, TN-C may promote Th17 expansion through its ability to induce Th17-inducing cytokine production from DCs and form an important link from innate to adaptive immunity.

**Figure 1:**
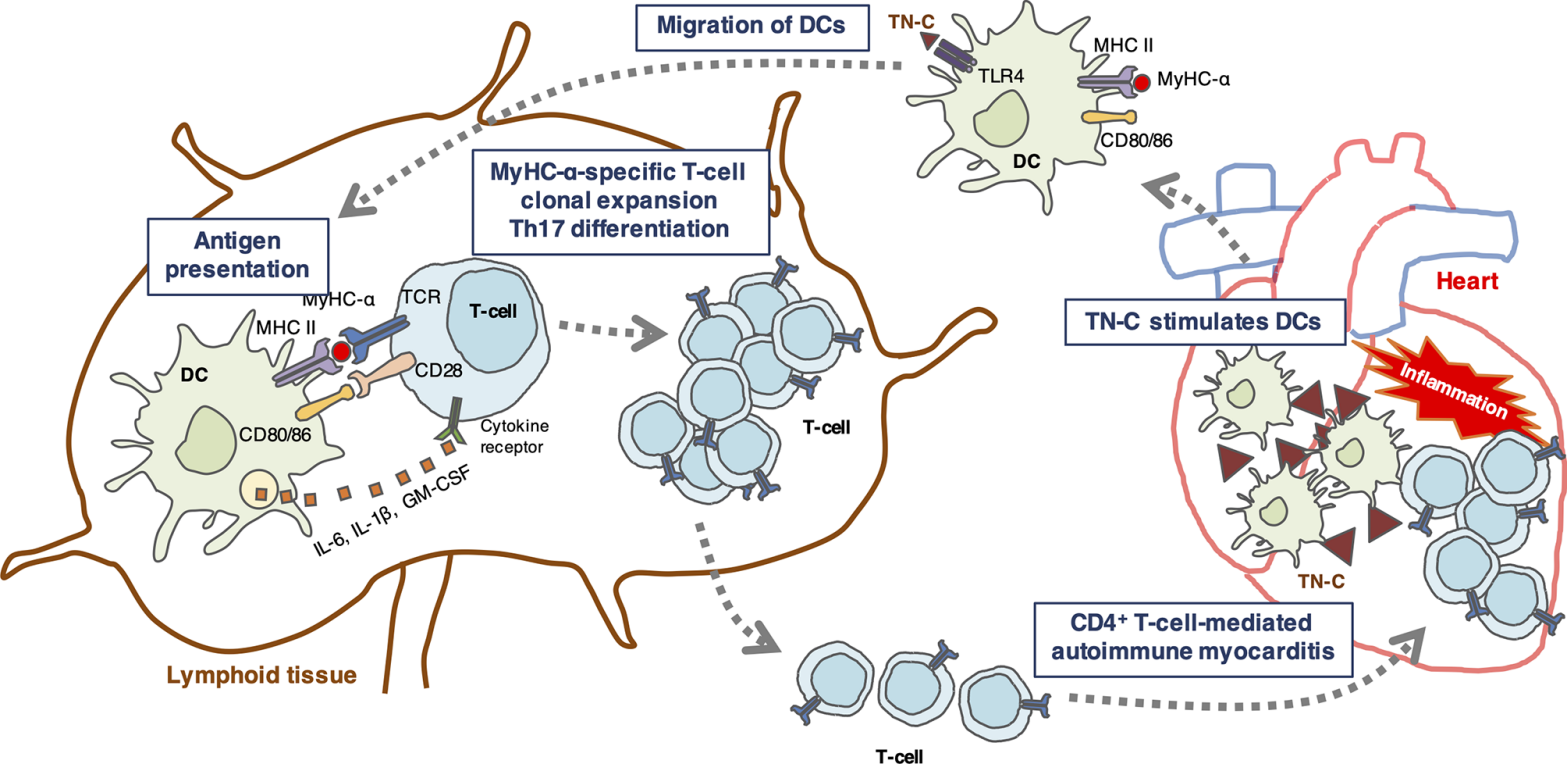
Schematic illustration showing a hypothetical mechanism by which tenascin-C (TN-C)-stimulated dendritic cells (DCs) induce Th17 differentiation. TN-C is upregulated in the heart under pathological conditions such as tissue injury and inflammation, and stimulates myocardial dendritic cells (DCs) *via* toll-like receptor 4 (TLR4) activation in the heart. Activated DCs migrate to the cardiac draining lymph nodes where they activate cardiac myosin-specific T cells. DCs produce Th17-polarizing cytokines (IL-6, IL-1, and GM-CSF) that contribute to the generation of Th17 cells. In turn, CD4^+^ T cells migrate back to the heart and cause autoimmune myocarditis.

DCs are antigen-presenting cells essential for priming T cell responses (61). Resting tolerogenic DCs that display cardiac myosin peptides in complex with class II major histocompatibility complex (MHC) are present in a healthy heart and are trafficked to the cardiac draining lymph nodes. In the lymph nodes, DCs present cardiac myosin peptides to naïve CD4^+^ T cells specific to those peptides, leading to deletion, anergy, or Treg induction (32). If a heart is damaged by tissue injury or inflammation, TN-C is produced by fibroblasts and stimulates myocardial DCs to migrate to the cardiac draining lymph nodes and activate cardiac myosin-specific T cells, which then differentiate into inflammatory effector T cells (**Figure 1**). In addition to presenting antigen-derived peptides on their MHCs with costimulatory molecules for naïve T cell activation and expansion, DCs release a cocktail of polarizing cytokines for the differentiation of CD4^+^ T cells into effector cells (30, 62). In autoimmune diseases, DCs play an important role in the regulation of autoreactive CD4^+^ T cells (30). A model of bone marrow-derived DC (BMDC)-induced autoimmune myocarditis is helpful for understanding how DCs activate autoreactive CD4^+^ T cells (28). In this model, the activation of TLRs on BMDCs loaded with a MyHC-α peptide is essential for the induction of autoimmune myocarditis (9, 28). TLR signaling triggers innate immunity upon stimulation with microbial products or endogenous danger signals (danger-associated molecular patterns [DAMPs]) (63). In sterile inflammation, DAMPs are released from either ECM (e.g., TN-C or biglycan) or from dying cells (e.g., histones, high mobility group box 1, heat-shock proteins, DNAs, or RNAs) and stimulate TLRs (64, 65). Popovic *et al.* reported that an endogenous TLR2/4 ligand biglycan enhanced the priming of autoreactive T cells and stimulated autoimmune perimyocarditis (66). We previously showed that TN-C provides DCs to induce myocarditis *via* TLR4 activation (9). The injection of MyHC-α-loaded BMDCs stimulated with TN-C induced myocarditis in the recipient mice (9). Upon stimulation with TN-C, DCs produced large amounts of proinflammatory cytokines, including the Th17-polarizing cytokines IL-1β, IL-6, and GM-CSF (9). Naïve CD4^+^ T cells co-cultured with TN-C-stimulated DCs differentiated into Th17 cells, but IL-6 blocking antibody inhibited Th17 polarization (9). Moreover, the blocking of TLR4 signaling reduced IL-6 secretion from DCs with less Th17 generation (9). TN-C-stimulated BMDCs from TLR4-deficient mice failed to induce myocarditis in the recipients, indicating that TN-C provides myocarditis inducibility to DCs, at least in part, *via* TLR4 interaction (**Figure 1**) (5, 9). However, this concept is based on limited experimental findings. Therefore, further studies are needed to fully determine the effect of TN-C on the onset and progression of myocarditis.

## Conclusions and Perspectives

The etiology and pathogenesis of myocarditis are not yet fully understood. TN-C may be a key extracellular modulator that controls immune responses in myocarditis and inflammatory cardiomyopathy. To date, no attempt has been made to suppress the function of TN-C during myocarditis; however, it has been reported that the administration of an antibody against a domain of TN-C to a rheumatoid arthritis model ameliorated disease severity. Thus, blocking TN-C-dependent inflammatory signals may be a potential novel therapeutic strategy for treating autoimmune myocarditis. Studies involving various animal models have provided a plethora of information, but there remains a gap in knowledge regarding how myocarditis in animal models may differ from that in humans. The prevalence of myocarditis will increase together with the expanding use of immune checkpoint inhibitors and the progression of the COVID-19 pandemic (67). As a result, sophisticated technologies, computational models, and insights are needed.

## Author Contributions

All authors listed have made a substantial, direct, and intellectual contribution to the work and approved it for publication. All authors contributed to the article and approved the submitted version.

## Funding

This study was supported in part by a Grant-in-Aid for Scientific Research (Japan Society for the Promotion of Science KAKENHI grant number 20K08396) to KT.

## Conflict of Interest

The authors declare that the research was conducted in the absence of any commercial or financial relationships that could be construed as a potential conflict of interest.
